# Evaluating the design of the Integrated Care for Older People: a theory of change approach

**DOI:** 10.3389/fmed.2023.1166196

**Published:** 2023-07-12

**Authors:** Samuel E. Gutiérrez-Barreto, Eduardo Sosa-Tinoco, Oscar Rojas-Calixto, Zayda Deniss-Navarro, Arturo Avila-Avila, Juan Pablo Gutierrez

**Affiliations:** ^1^Master’s and Doctorate Programs in Medical, Dental, and Health Sciences, National Autonomous University of Mexico, Mexico City, Mexico; ^2^National Institute of Geriatrics, Mexico City, Mexico; ^3^Ministry of Health, Mexico City, Mexico; ^4^Center for Policy, Population and Health Research, National Autonomous University of Mexico, Mexico City, Mexico

**Keywords:** ICOPE, design evaluation, theory of change, community health services, older people

## Abstract

**Introduction:**

Given the progressive aging of the population, there is an urgent need at the health system level to implement effective models to care for older people (OP). Healthy aging is imperative to reach the Sustainable Development Goals. The World Health Organization (WHO) developed the Integrated Care for Older People (ICOPE) strategy to address this challenge. Implementing ICOPE requires its adaption to a specific context. We propose a pathway for such adaptation through an evaluation of the design of ICOPE; thus, we aim to describe the Theory of Change (ToC) of ICOPE and evaluate it for its implementation in Mexico City.

**Methods:**

Based on the WHO and published literature documentation, we drafted an initial ToC for ICOPE. Then, we validated the ToC with experts in ICOPE, after which we evaluated and refined it by discussing the causal pathway, intervention required to activate it, rationale, and assumptions in consecutive workshops with 91 stakeholders and healthcare workers, using the nominal group technique to reach a consensus.

**Results:**

The resulting ToC has the potential to contribute to healthy aging by three expected impacts: (1) prevention, reversal, or delaying of the decline of intrinsic capacity (IC) in OP; (2) improvement of the quality of life of OP; and (3) increase of disability-free life expectancy. The ICOPE causal pathway had ten preconditions, including the availability of resources, identifying at-risk individuals, available treatments, and evaluating results.

**Discussion:**

We adapted ICOPE to a specific implementation context by evaluating its ToC in a participatory process that allows us to identify challenges and address them, at least in terms of the guidelines to operate the strategy. As ICOPE is an approach for a primary healthcare system, its adoption in a community healthcare program is promising and feasible. Evaluation as a tool could contribute to the design of effective interventions. The evaluation of the design of ICOPE for its implementation contributes to the strength of its potential to improve care for OP. This design for implementing ICOPE has the potential to be applied to similar contexts, for example, in other lower-middle-income countries.

## Introduction

According to the World Health Organization (WHO), by 2050, more than 1 in 5 people will be 60 years or older (1), a population group with increasing needs in terms of healthcare. Older people (OP) utilize more health services than younger adults, usually with a larger share of specialized care (2, 3). In Mexico, OP currently represent 14% of the total population, a share that will almost double by 2050. Mexico is just below the United States, where the current share of OP is 16.2%. As the proportion of the OP increases, healthcare models must adapt their approach to fulfill their needs.

Existing healthcare models in general were developed based on a different population profile, and their adaption to the specific needs of OP is not straightforward (4, 5). Aligned with the Sustainable Development Goals, the United Nations declared the current decade of the 2020s as the decade of Healthy Aging as a strategy for achieving and supporting actions to build a society for all ages (6), inclusive for OP and avoiding so-called ageism, i.e., discrimination based on age.

The WHO developed the Integrated Care for Older People (ICOPE) approach to strengthen how existing healthcare models provide care for OP, which focuses on preventing decline or loss and restoring individual intrinsic capacity (IC). IC is the composite of all physical and mental capacities an individual can draw (7). The implementation of the ICOPE approach includes 19 actions classified into essential and non-essential and further categorized into three levels: (i) macro (e.g., strengthen governance and accountability systems), (ii) meso (e.g., orient services towards primary care), and (iii) micro (e.g., guidelines for dimensions on IC) (8). For the micro level, ICOPE comprises guidance on person-centered assessment with six guides for multiple types of healthcare workers (9).

These multiple components acting independently and in conjunction with the health system operation make the ICOPE approach complex. The WHO developed the ICOPE approach for its implementation worldwide, requiring further adaptation to each specific country or subnational area. This adaptation involves how the proposed actions align with the existing healthcare model and the refinements required. An evaluation of the design of ICOPE is a promising approach to identify such refinements, as it could identify potential limitations and challenges to accomplish the desired outcome and thus distill the intervention to increase its potential. That is, analyzing the implicit Theory of Change (ToC) of ICOPE and refining it by evaluating it. The ToC approach is a tool that helps to identify how an intervention expects to reach its long-term outcomes through a logical sequence of intermediate outcomes (10, 11). It has extensive applications, reported in the literature, to evaluate and design healthcare interventions (11). Several studies have demonstrated the extra benefits of the model of action and the unforeseen consequences of the intervention (12). A ToC evaluation can effectively assess the expected mechanism through which the intervention could produce a change and how the context may modulate these effects. The ICOPE approach can profit from the design evaluation as it will inform how to adapt it to a specific context. Therefore, this study aims to evaluate the design of the ICOPE by making its ToC explicit and assess it from the perspective of implementing ICOPE within a primary care health program in Mexico City.

## Materials and methods

This evaluative study used a documental review and qualitative tools to draft and assess the ToC model of ICOPE for its implementation within a primary care program in Mexico City. The provision of health services in Mexico is segmented by population labor condition, with 40.4% of the population covered by the social security services that provide care for formal employees and their families, 16.1% by the private sector, and 43.5% by public services (13).

### Setting

We analyzed the potential of ICOPE in Iztacalco, one of Mexico City’s 16 boroughs. Iztacalco had a population of 404,695 people in 2020; around 16% were aged 60 years or above (14). About 37% of that population was attending local public health services, while the remaining population was receiving care from social security (51%) or private (12%) subsystems (14). Iztacalco is a municipality with 25.2% of the population living in poverty and 25.4% reporting lack of access to health services (14). Public health services for OP in Iztacalco without social security comprise five primary health facilities, one geriatric clinic, and a healthcare program to provide health services in their homes. This health program, called “*Salud en tu Casa*,” is staffed by general physicians, nurses, dentists, health officers, physiotherapists, nutritionists, psychologists, and social workers. None of them has formal training to provide health services, promotion, and prevention activities to OP. These healthcare workers (HCWs) provide health services for around 5,000 OP in their residences and liaise with them in other government programs.

### Drafting the theory of change of ICOPE and ToC workshop participants

To draft a ToC for ICOPE, a documental review was implemented, focusing on the official publications from the WHO. Initially, we met with two stakeholders from the National Institute of Geriatrics in Mexico, who had extensive experience in the ICOPE approach to further review the initial draft and ensure it reflected the scope of the strategy. Two workshops were conducted in 2022 to evaluate the design of ICOPE for its implementation in Iztacalco within *Salud en Tu Casa*. The first was with five persons, directors, and stakeholders from the healthcare program and the geriatric clinic from Iztacalco. In the second workshop, 82 persons participated; they were HCWs from Iztacalco. All the participants had diverse professional backgrounds (geriatrics, public health, primary care, education, nursing, social work, nutrition, psychology, physiotherapy, and dentistry).

### Procedures

The evaluation of the design of ICOPE underwent two stages: (a) drafting and validating of an initial ToC and (b) evaluating and redefining of the ToC. The first stage implicated a provisional ToC development using a literature review and a meeting with a structured discussion about revising the scope of ICOPE. This discussion was conducted in a videoconference in February 2022 facilitated by both authors (SEGB and JPG). For the evaluation in the second stage, we conducted two workshops in Iztacalco borough in April 2022. Two researchers, experts in program evaluation and ToC, facilitated the workshops. During the workshops, both facilitators (AAA and SEGB) emphasized that the focus of involvement with the ToC components for some participants went beyond the HCW duty. Moreover, we established the work’s objectives, provided a brief description of the ToC approach, and used the nominal group technique to reach an agreement. We used the approach suggested by Breuer and collaborators to develop and report the ToC (11, 15). The first step recommended to validate the ToC during the workshops was the definition of the impacts and long-term outcomes. Then, we iteratively worked backward to map out the preconditions, interventions, assumptions, and indicators to generate the desired outcomes (15). The process used a multi-voting system to reach a consensus on each element, and in case of disagreement, we performed a guided discussion.

In evaluating the design of ICOPE, we refined the ToC, considering the written feedback and expert consultation on the program evaluation. Then, in a meeting with the stakeholder group (two geriatricians and two program health directors), we discussed and redefined the ToC. This structured discussion was held at the National Institute of Geriatrics in Mexico City in June 2022. After a presentation and recap of the ToC process and existing ToC map, the group discussed the practical problems encountered in the borough, e.g., the specific context barriers.

### Data collection

We searched for published documents that described the features and characteristics of the ICOPE approach. From this search, we identified two primary documents (8, 16) that described four main categories of ICOPE: (1) resources needed, (2) ability to identify at-risk individuals, (3) available treatments, and (4) long-term outcomes. For the workshops, the first author collected the ToC workshops’ data *via* audio-recorded real-time notes. The two workshops lasted 2:00 h and 1:30 h, respectively. We have used the documental review and workshop data to evaluate the ICOPE design and describe the ToC for its implementation in Mexico City.

## Results

**Figure 1:**
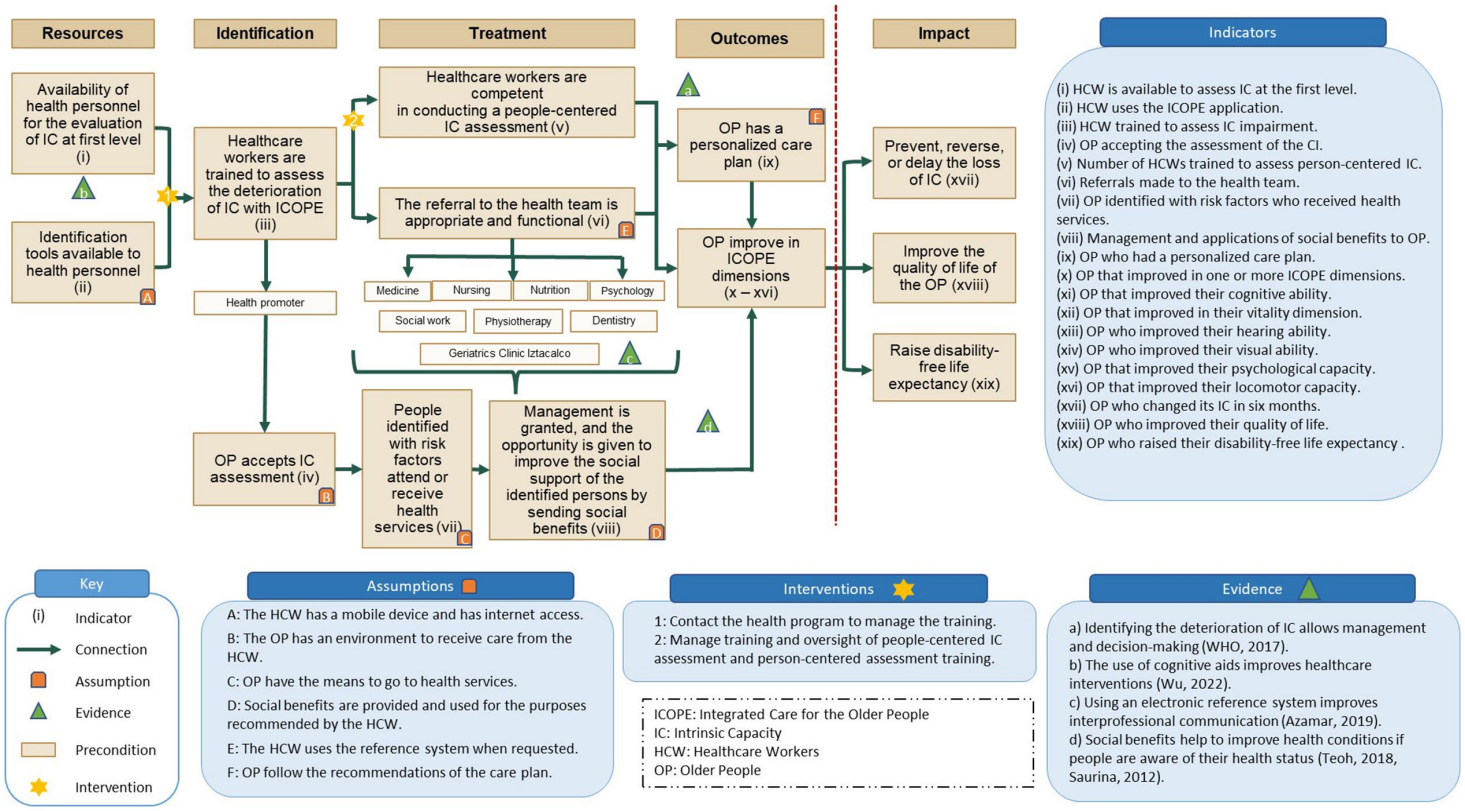
Theory of Change of the Integrated Care for Older People in Salud en tu Casa.

Table 1 describes the characteristics of the 91 HCWs and stakeholders’ participants at the meeting and the two ToC workshops. In the following sections, we detailed (1) the finally agreed ToC with the main elements (Figure 1) and (2) the findings from the evaluation of the design of the ICOPE approach, including the narrative of the ToC with its preconditions, assumptions, interventions, and rationales.

**Table 1:** Participants in the development of the ToC workshops.

Participants	*N*	Females
*Structured discussion*	4	0 (0%)
Geriatricians	2	0
Researchers	2	0
*ToC 1*	5	3 (60%)
Stakeholders	2	0
Administrator	1	1
Nurse	1	1
Dentist	1	1
*ToC 2*	82	56 (68%)
Geriatricians	2	2
Physicians	14	9
Nurse	16	15
Health promoter	20	10
Stakeholders	3	0
Social worker	4	3
Administrator	9	7
Psychologist	4	2
Nutritionist	2	2
Audiologist	1	1
Physiotherapist	3	2
Dentist	4	3

While we found that all participants supported using the ICOPE approach (to which they were previously exposed in training), they also expressed their need to gain experience in its application by using the guidelines to provide care services. From the HCWs perspective, critical barriers to activating the causal pathway included limited supervision and feedback in their daily work, lack of collaboration between the HCWs’ to provide integrated care, and technical issues using cognitive aids such as mobile applications to assess OP. All HCWs recognize the need for more courses to provide better care for OP. Also, they realized they would need a system to consult with a geriatrician or other specialist if they could not give further care to the OP. The findings of this study acknowledged that the development of the ToC, particularly to triangulate all the components, requires external stakeholders that support the project; that is, more is needed for the staff.

### Long-term outcomes and impacts

In the literature review, the researchers proposed that the long-term outcome was that (i) OP would have a personalized care plan. In the first workshop, as the second long-term outcome, the participants added (ii) OP would improve in ICOPE dimensions. Concerning the impacts in the literature review, three impacts were established, and through the workshops, those remained as (i) preventing, reversing, or delaying the loss of intrinsic capacity (IC), (ii) improve the quality of life of OP, and (iii) raising disability-free life expectancy. The HCWs expressed concerns about the long-term outcomes and impacts. The main concern was the lack of a guideline that specifies (a) how to prioritize the treatments needed for OP with deficits of one or more dimensions, (b) how to reference OP needing to visit the geriatric clinic, and (c) the referral and back-referral mechanisms for OP between all the HCWs. To help tackle these concerns, the stakeholders in the last redefinition of the ToC proposed using a procedural manual and mobile app to conduct the follow-up. An additional concern was related to the feasibility of the defined impacts, as for some HCWs, only some of the dimensions of the ICOPE would produce a change.

The positive changes in all the workshops were that using the ICOPE approach would positively impact the daily work of the HCWs. The stakeholders and HCWs identified the need for interventions and several preconditions to achieve long-term outcomes and contribute to the impacts.

### Narrative of the ToC

The expected long-term outcomes are that (i) OP have personalized care plans and (ii) they improve in the ICOPE dimensions; the expected impact is the improvement in IC (17, 18). The outcomes imply that OP will change their health-related behaviors based on the recommendations of their personalized care plans. This outcome will modify some of the IC’s dimensions, e.g., improving their locomotion by using or receiving mobility aids, such as a cane. The long-term outcome indicators are time constrained and depend on the behavior of OP.

### Interventions

The first intervention marked in Figure 1 as a star is the reach of the health program to manage the training. Before the first interventions and to activate the causal pathway of the ToC program, two preconditions are required: (1) availability of health personnel for the evaluation of IC at the first level and (2) availability of identification tools to health personnel. The second precondition assumes that the HCWs have access to an internet connection and a mobile device. As a rationale, there is evidence that using cognitive aids improves healthcare interventions (19). The indicators of both preconditions are that (i) a HCW is available to assess IC at the first level and (ii) a HCW uses the ICOPE application.

The second intervention is managing training and the supervision of people-centered IC and person-centered assessment training. The preconditions before this intervention were that (3) healthcare workers were trained to assess the deterioration of IC with ICOPE and (4) OP would accept IC assessment. The fourth precondition assumes that OP have an environment to receive care from HCWs. During the third workshop, the health promoters mentioned that OP sometimes need a proper place to receive healthcare in their residences.

Furthermore, in the workshop, they proposed explicitly identifying within the causal pathway the composition of the team responsible for providing healthcare at the different levels of diagnosis and treatment. The indicators for these preconditions are that (iii) HCW are trained to assess IC impairment and (iv) OP accept the assessment of the CI.

At the “Treatment” level, the preconditions are that (5) healthcare workers are competent in conducting a people-centered IC assessment, (6) the referral to the health team is done appropriately and functionally, (7) people identified with risk factors attend or receive health services, and (8) management and opportunity are given to improve the social support of the identified persons by sending them social benefits. The referral system’s precondition depends on using the existing system when requested. This assumption was discussed during the third workshop by the HCWs, who expressed concerns about the reference system’s lack of supervision. The rationale for using an electronic reference system is the evidence supporting its use to increase interprofessional communication and leadership (20). The sixth and seventh preconditions assume that the other social programs offered to OP are used for the purposes recommended by HCWs and that OP have the means to attend health services. The stakeholders discussed these preconditions in the second workshop; the central comment was the motivation of HCWs to provide care and of OP to use the available resources to improve their health. The rationale is that social benefits improve health conditions if individuals know their health status (21, 22). The indicators for the preconditions were (v) the number of HCWs trained to assess person-centered IC, (vi) referrals made to the health team, (vii) OP identified with risk factors who received health services, and (viii) management and applications of social benefits to OP.

## Discussion

By evaluating the design of ICOPE for its implementation in Mexico City using a Theory of Change approach, we have produced a change model that could contribute to the wellbeing of OP if implemented accordingly.

During this evaluation, we identified a set of attributes required to strengthen the design of ICOPE. Our refined ToC has the potential to achieve the expected outcomes of ICOPE in Iztacalco. It describes the required interaction between HCWs and OP in Iztacalco and explicitly states that both the demand and supply sides are needed to achieve ICOPE’s goals (12, 23–27).

Using an evaluative approach, we were able to identify a feasible pathway in the Iztacalco context of *Salud en Tu Casa* to reach the long-term outcomes that are expected to ultimately contribute to (1) reversing, preventing, or delaying the loss of IC, (2) improving the quality of life of OP, and (3) raising disability-free life expectancy. Drafting the validated ToC was possible through a participatory process with key stakeholders and the HCWs that critically discussed the intermediate outcomes, assumptions, and rationale for ICOPE, with similar results reported by the WHO in their ready phase study (28).

The ToC consists of four significant levels, namely, resources, identification, treatment, and long-term outcomes, to activate the causal pathway. The identification and treatment levels are equivalent to the activities reported in other ToC developments (11). These involve the essential activities proposed by the WHO implementation framework (4, 29). The causal pathway explicitly articulates how the community healthcare program would provide care to OP to achieve sustainable change (30). One of the examples was that the external stakeholders identified the social benefits that OP could benefit from at the treatment level with less caregiver support.

During the workshops, the HCWs discussed the likelihood of implementing ICOPE within Salud en tu Casa with the ToC and redefined their daily work. They also discussed and agreed on some of the resources needed for the operation of the ToC, like other studies (28, 31). As previously discussed, resource constraints have been a significant barrier to improving healthcare services in Mexico and lower-middle-income countries (LMIC) (2). Moreover, the accomplishment of the preconditions could increase the motivation of HCWs and enhance their delivery of quality care. ICOPE, as operationalized in the developed ToC, relies on the available resources for a community-based health program already operating in Mexico City, which is feasible. At the resources level, using a mobile app to run the ICOPE approach has presented several benefits (32–34). The discussions with stakeholders in the redefinition stage were valuable in overseeing the barriers mentioned in the previous workshops. For example, the directors must supervise the health promoter using the screening evaluation guide (9). In addition, we established the necessity of giving the promoters headphones to apply the audition test carefully. The screening tool used in ICOPE had good sensitivity but depended on the training (35), so the directors recognized the necessity of the continuous training of HCWs. A key element for the success of new interventions is to ensure the buy-in from the relevant stakeholders (36); the participatory process used in evaluating and refining the ToC contributes to this by generating a sense of ownership of the approach. At the ToC treatment level, the healthcare program has all the disciplines of the six domains for the ICOPE approach. The main barrier discussed was integrating the services between the levels of care and the system to collect the indicators’ data (the community health program and the geriatrics clinic). The proposal of a straightforward approach in the ToC and improved communication with a mobile application could guide the clinical pathway to provide health services for older adults. Using an electronic referral system improves interprofessional communication and services (20).

## Conclusion

The long-term outcomes of the ToC regarding IC were coherent with the ICOPE program goal of healthy aging. After evaluating its design and further refinement of its ToC, ICOPE implementation in a community healthcare program has been shown to be promising and feasible. The results could contribute to monitoring the trajectory of IC and its domains. The specific interventions of the model were found to be possible to implement by the relevant stakeholders and personnel in charge of the operation. The design evaluation of ICOPE in the community healthcare program showed evidence of validity for improving clinical care management for OP. This strategy for implementing ICOPE has the potential to be applied in similar contexts, for example, other LMICs.

## Data availability statement

The raw data supporting the conclusions of this article will be made available by the authors, without undue reservation.

## Ethics statement

The studies involving human participants were reviewed and approved by Ethics Committee and Institutional Review Board of the Masters and Doctorate Program in Medical, Dental, and Health Sciences, the National Autonomous University of Mexico Program. The patients/participants provided their written informed consent to participate in this study.

## Author contributions

SEG-B and JP-G: conceptualization, study design, data interpretation, original draft preparation, project administration, reviewing, and editing. ES-T and AA-A: data acquisition, interpretation, original draft preparation, project administration, and reviewing. OR-C and ZD-N: data acquisition, interpretation, original draft preparation, reviewing, and editing. All authors contributed to the article and approved the submitted version.

## Conflict of interest

The authors declare that the research was conducted in the absence of any commercial or financial relationships that could be construed as a potential conflict of interest.

## Publisher’s note

All claims expressed in this article are solely those of the authors and do not necessarily represent those of their affiliated organizations, or those of the publisher, the editors and the reviewers. Any product that may be evaluated in this article, or claim that may be made by its manufacturer, is not guaranteed or endorsed by the publisher.

## References

[1] World Health Organization. Global strategy and action plan on aging and health [Internet]. Geneva, Switzerland WHO; (2017) 1–56

[2] Kalseth J Halvorsen T. Health, and care service utilization and cost over the lifespan: a descriptive analysis of population data. BMC Health Serv Res. (2020) 20:435. doi: 10.1186/s12913-020-05295-2, PMID: 32429985 PMC7236310

[3] Gutiérrez Robledo LM Kershenobich Stalnikowitz D. Envejecimiento y salud: una propuesta para un plan de acción [Internet]. Available at: http://www.geriatria.salud.gob.mx/descargas/publicaciones/Envejecimiento_y_salud_3a_edicion.pdf

[4] de Carvalho IA Epping-Jordan JA Pot AM Kelley E Toro N Thiyagarajan JA. Organizing integrated healthcare services to meet older people’s needs. Bull World Health Organ. (2017) 95:756–63. doi: 10.2471/BLT.16.187617, PMID: 29147056 PMC5677611

[5] World Health Organization. Informe mundial sobre el envejecimiento y la salud [Internet]. (2015). Available at: https://apps.who.int/iris/bitstream/handle/10665/186466/9789240694873_spa.pdf;jsessionid=9ECA2F3B1BD0A4EAAB9F8B11E3EBD33B?sequence=1

[6] United Nations. Transforming our world: The 2030 agenda for sustainable development. San Francisco: United Nations (2015).

[7] World Health Organization. Integrated care for older people guidelines on community-level interventions to manage declines in intrinsic capacity. Geneva: World Health Organization (2017). 29608259

[8] World Health Organization. Implementation framework guidance for systems and services. (2019). 1–41 p.

[9] World Health Organization. *Guidance on person-centered assessment and pathways in primary care*. Handbook [Internet]. (2019). Available at: https://apps.who.int/iris/bitstream/handle/10665/326843/WHO-FWC-ALC-19.1-eng.pdf?sequence=17&isAllowed=y%0Ahttps://apps.who.int/iris/bitstream/handle/10665/326843/WHO-FWC-ALC-19.1-eng.pdf?sequence=17

[10] Vogel I. Review of the use of ‘Theory of Change’ in international development [Internet]. (2012). Available at: www.isabelvogel.co.uk

[11] Breuer E Lee L de Silva M Lund C. Using theory of change to design and evaluate public health interventions: a systematic review. Implement Sci. (2016) 11:1–17. doi: 10.1186/s13012-016-0422-6, PMID: 27153985 PMC4859947

[12] Arensman B van Waegeningh C van Wessel M. Twinning “practices of change” with “theory of change”: room for emergence in advocacy evaluation. Am J Eval. (2018) 39:221–36. doi: 10.1177/1098214017727364, PMID: 30886515 PMC6377054

[13] Block MÁG Reyes H Lucero M Hurtado C Balandrán A Méndez E. Health Systems in Transition Mexico Health system review, vol. 22 (2020). 2020 p Available at: www.healthobservatory.eu. 33527902

[14] Instituto Nacional de Estadística y Geografia. INEGI [Internet] (2020). Available at: https://www.inegi.org.mx/programas/ccpv/2020/

[15] de Silva MJ Breuer E Lee L Asher L Chowdhary N Lund C. Theory of change: a theory-driven approach to enhance the Medical Research Council’s framework for complex interventions. Trials. (2014) 15:1–12. doi: 10.1186/1745-6215-15-267 24996765 PMC4227087

[16] Briggs AM De CIA. Actions required to implement integrated care for older people in the community using the World Health Organization s ICOPE approach: a global Delphi consensus study. PLoS One. (2018) 13:e0205533. doi: 10.1371/journal.pone.0205533 30308077 PMC6181385

[17] Zhou Y Ma L. Intrinsic capacity in older adults: recent advances. Aging Dis. (2022) 13:353–9. doi: 10.14336/AD.2021.0818, PMID: 35371613 PMC8947834

[18] Cesari M De Carvalho IA Thiyagarajan JA Cooper C Martin FC Reginster JY. Evidence for the domains supporting the construct of intrinsic capacity. In: Journals of gerontology - series a biological sciences and medical sciences. United Kingdom, Oxford, England: Oxford University Press (2018). 73:1653–60. 29408961 10.1093/gerona/gly011

[19] Wu P Zhang R Luan J Zhu M. Factors affecting physicians using mobile health applications: an empirical study. BMC Health Serv Res. (2022) 22:24. doi: 10.1186/s12913-021-07339-7, PMID: 34983501 PMC8729011

[20] Azamar-Alonso A Costa AP Huebner LA Tarride JE. Electronic referral systems in health care: a scoping review. Clin Econ Outcomes Res. (2019) 11:325–33. doi: 10.2147/CEOR.S195597, PMID: 31190925 PMC6511625

[21] Teoh AN Hilmert C. Social support as a comfort or an encouragement: a systematic review on the contrasting effects of social support on cardiovascular reactivity. Br J Health Psychol. (2018) 23:1040–65. doi: 10.1111/bjhp.12337, PMID: 30084181

[22] Saurina C Vall-Llosera L Saez M. Factors determining access to and use of primary health care services in the Girona Health Region (Spain). Eur J Health Econ. (2012) 13:419–27. doi: 10.1007/s10198-011-0313-3, PMID: 21499790

[23] Abayneh S Lempp H Alem A Kohrt BA Fekadu A Hanlon C. Developing a theory of change model of service user and caregiver involvement in mental health system strengthening in primary health care in rural Ethiopia. Int J Ment Health Syst. (2020) 14:51. doi: 10.1186/s13033-020-00383-6, PMID: 32760440 PMC7379363

[24] De-Silva M Ryan G. Using theory of change in the development, implementation, and evaluation of complex health interventions. Mental health innovation network (2015).

[25] Gilissen J Pivodic L Gastmans C Vander Stichele R Deliens L. How to achieve the desired outcomes of advance care planning in nursing homes: a theory of change. BMC Geriatr. (2018) 18:1–14. doi: 10.1186/s12877-018-0723-5 29444645 PMC5813418

[26] Breuer E de Silva MJ Shidaye R Petersen I Nakku J Jordans MJD. Planning and evaluating mental health services in low-and middle-income countries using theory of change. Br J Psychiatry. (2016) 208:s55–62. doi: 10.1192/bjp.bp.114.153841, PMID: 26447178 PMC4698557

[27] DuBow WM Litzler E. The development and use of a theory of change to align programs and evaluation in a complex national initiative. Am J Evaluat. (2019) 40:231–48. doi: 10.1177/1098214018778132

[28] World Health Organization. ICOPE: findings from the ‘ready’ phase implementation pilot program. Geneva: World Health Organization (2022).

[29] Organización Mundial de la Salud. Orientación para los sistemas y servicios: marco de aplicación de ICOPE. Geneva: Organización Mundial de la Salud (2020).

[30] Koleros A Mulkerne S Oldenbeuving M Stein D. The actor-based change framework: a pragmatic approach to developing program theory for interventions in complex systems. Am J Eval. (2020) 41:34–53. doi: 10.1177/1098214018786462

[31] Tavassoli N Piau A Berbon C de Kerimel J Lafont C de Souto BP. Framework implementation of the INSPIRE ICOPE-CARE program in collaboration with the World Health Organization (WHO) in the Occitania region. J Frail Aging. (2020) 10:1–7. doi: 10.14283/jfa.2020.26, PMID: 33575698

[32] Sanchez-Rodriguez D Piccard S Dardenne N Giet D Annweiler C Gillain S. Implementation of the integrated care of older people (ICOPE) app and ICOPE monitor in primary care: a study protocol. J Frailty Aging. (2021) 10:1–7. doi: 10.14283/jfa.2021.22 34105715

[33] Barreto S. Person-centered intervention: the ICOPE experience in France coordinator of the institute on aging-toulouse gérontopôle databa se ICT tools (n.d.).

[34] Tavassoli N de Souto BP Berbon C Mathieu C de Kerimel J Lafont C. Implementation of the WHO integrated care for older people (ICOPE) program in clinical practice: a prospective study. Lancet Healthy Longev. (2022) 3:e394–404. doi: 10.1016/S2666-7568(22)00097-6, PMID: 36098317

[35] Ma L Chhetri JK Zhang Y Liu P Chen Y Li Y. Integrated care for older people screening tool for measuring intrinsic capacity: preliminary findings from ICOPE pilot in China. Front Med (Lausanne). (2020) 7:576079. doi: 10.3389/fmed.2020.576079 33330532 PMC7734133

[36] Wu S Tannous E Haldane V Ellen ME Wei X. Barriers, and facilitators of implementing interventions to improve appropriate antibiotic use in low- and middle-income countries: a systematic review based on the consolidated framework for implementation research. Implement. Sci. (2022) 17:30. doi: 10.1186/s13012-022-01209-4 35550169 PMC9096759

